# Sono-Chemical Synthesis of Silver Quantum Dots Immobilized on Exfoliated Graphitic Carbon Nitride Nanostructures Using Ginseng Extract for Photocatalytic Hydrogen Evolution, Dye Degradation, and Antimicrobial Studies

**DOI:** 10.3390/nano11112918

**Published:** 2021-10-31

**Authors:** Koduru Mallikarjuna, Surya Veerendra Prabhakar Vattikuti, Ravi Manne, Gangarapu Manjula, Keelapattu Munirathnam, Srinivas Mallapur, Najat Marraiki, Arifullah Mohammed, Lebaka Veeranjaneya Reddy, Megala Rajesh, Mohammad Khairul Azhar Abdul Razab

**Affiliations:** 1 Department of Physics, Siddharth Institute of Engineering and Technology, Puttur 517583, India; mallikar999@gmail.com; 2 School of Mechanical Engineering, Yeungnam University, 280 Daehak-ro, Gyeongsan-si 38533, Gyeongsangbuk-do, Korea; vsvprabu@gmail.com; 3 Chemtex Environmental Lab, Port Arthur, TX 77642, USA; ravimannemr@gmail.com; 4 Department of Physics, Sri Venkateswara College of Engineering, Tirupati 517520, India; manjulasvu2@gmail.com; 5 Department of Physics, School of Applied Sciences, REVA University, Bangalore 560064, India; mail2rathnam@gmail.com; 6 Department of Chemistry, School of Applied Sciences, REVA University, Bangalore 560064, India; seenuseenum@gmail.com; 7 Department of Botany and Microbiology, College of Science, King Saud University, P.O. 2455, Riyadh 11451, Saudi Arabia; najat@ksu.edu.sa; 8 Faculty of Agro-Based Industry, Universiti Malaysia Kelantan Campus Jeli, Locked Bag100, Jeli 17600, Kelantan, Malaysia; 9 Department of Microbiology, Yogi Vemana University, Kadapa 516003, India; 10 Department of Physics, Sri Venkateswara University, Tirupati 517502, India; megalarajesh999@gmail.com; 11 School of Health Sciences, Universiti Sains Malaysia, Health Campus, Kubang Kerian 16150, Kelantan, Malaysia

**Keywords:** ultrasonication, silver quantum dots, exfoliation g-C_3_N_4_, visible catalyst, dye degradation, H_2_ production, antibacterial studies

## Abstract

Due to modernization and the scarcity of fossil fuel resources, energy demand is continuously increasing. In this regard, it is essential and necessary to create a renewable energy source that can meet future energy demands. Recently, the production of H_2_ by water splitting and removing pollutants from the water has been essential for issues of energy and environmental demands. Herein, g-C_3_N_4_ and Ag-g-C_3_N_4_ composite structures have been successfully fabricated by the ultrasonication method. The physio/photochemical properties of prepared g-C_3_N_4_ and Ag-g-C_3_N_4_ were examined with different analytical techniques such as FTIR, XRD, UV-DRS, SEM, TEM, PL, and XPS analyses. The silver quantum dots (QDS) anchored to g-C_3_N_4_ structures performed the profound photocatalytic activities of H_2_ production, dye degradation, and antimicrobial activity under visible-light irradiation. The Ag/g-C_3_N_4_ composite with an Ag loading of 0.02 mole has an optimum photoactivity at 335.40 μmol g^−1^ h^−1^, which is superior to other Ag loading g-C_3_N_4_ composites. The synthesized Ag/g-C_3_N_4_ nanoparticles showed potential microbial inhibition activity during the preliminary screening, and the inhibition zones were comparable to the commercial antibiotic chloramphenicol. The loading of Ag into g-C_3_N_4_ paves the suppression, recombination and transfer of photo-generated electron-hole pairs, leading to the enhancement of hydrogen production, the diminishment of pollutants in water under visible light irradiation, and antimicrobial activity against multidrug-resistant pathogens.

## 1. Introduction

In recent decades, the entire world has been focused on environmental pollution and energy crises [1,2]. To date, several methods have been adopted to treat the degradation of untreated dyes in polluted wastewater, since they are highly active to light that can be obtained from the sun, and also because it is a simpler method that has a strong oxidizing ability and is environmentally friendly and energy saving [3,4]. Photocatalysis is one of the most promising methods to combat the challenges of dye degradation and water splitting for hydrogen fuel production, as well as their uses in medicinal applications [5,6]. Particularly in photocatalysis, the photocatalyst materials must have a high efficiency of utilization and conversion of solar light, more active photocatalytic facets, and a broad absorption spectrum [7,8,9]. Graphitic carbon nitrate (g-C_3_N_4_) is recognized as one of the metal-free photocatalysts that has unique characteristics; it is non-toxic, easy to prepare, has a desirable bandgap in the visible region, and is low-cost [10,11,12,13,14]. Recently, research on photocatalysis activity of g-C_3_N_4_ has become a more interesting topic due to its bandgap (2.7 eV) that responds in the visible light region, high adsorption capacity, large specific surface area, and good electron conductivity [15,16,17,18,19]. These properties enable the use of g-C_3_N_4_ in various fields such as fuel cell technology, CO_2_ photoreduction, solar cells, and water splitting [20,21,22,23,24]. However, pure g-C_3_N_4_ materials show low photocatalytic activity because of their high recombination rate of photoinduced mobile charge carries of electrons and holes [25]. However, the photocatalytic activities of g-C_3_N_4_ based materials were enhanced by metal (Ag, Au, Pt, and Pd) deposition, and added with other semiconductors [26]. Among all metals, silver is highly focused for deposition on g-C_3_N_4_ materials because of their unique chemical and physical properties and low cost [27].

In the present work, pure g-C_3_N_4_ and Ag QDs were deposited on exfoliated g-C_3_N_4_ nanocomposites and were prepared by sonochemical methods with ginseng extract. The effect of Ag content influences light absorbency of g-C_3_N_4_ and prolongs the lifetime of the photo-induced electron pair for hydrogen evolution and dye degradation under visible light. Moreover, their primary medicinal application to antimicrobial properties was investigated. 

## 2. Materials and Methods

### 2.1. Preparation Methods

**Figure 1:**
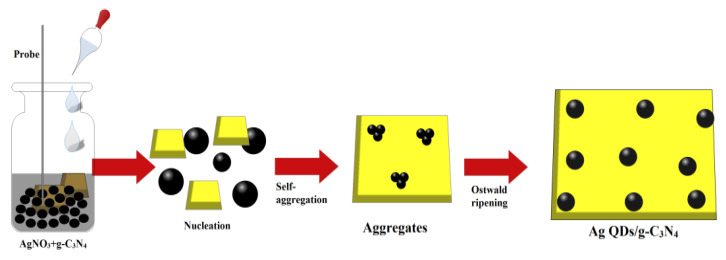
Growth mechanism of the formation of Ag QDs anchored to exfoliated g-C_3_N_4_ structures.

For the preparation of pure g-C_3_N_4_, 2 g of melamine was positioned on the oven to apply the heat of 450 °C for 2 h with a ramp rate of 2 °C/min. The attained sample was gathered and rinsed with ethanol to remove the unreacted compound. We prepared 5 mL of aqueous extract from ginseng root extract with 100 mg of g-C_3_N_4_ supported silver quantum dots by treating 0.005, 0.01, 0.015, 0.02, and 0.025 M of AgNO_3_ in 50 mL of distilled water under ultrasonic vibration with a frequency and power of 40 kHz and 150 W for 30 min at room temperature. The sonochemical procedure has significant advantages due to its simple and easy process to exfoliate the stacks to layers of bulk two-dimensional structures. The reaction transition to a grey color affirmed the formation of Ag/g-C_3_N_4_ suspension. The whole solution turned a grey color, suggesting the complete reduction of AgNO_3_ to Ag/g-C_3_N_4_, which is stable at room temperature. Fresh Korean ginseng roots were collected from a local market in Gyeongsan-si, South Korea. The roots were washed with distilled water to remove any dust materials. The ginseng root (100 g) was placed into a 1000 mL beaker with 500 mL of distilled water. The mixture was heated at 90 °C for 40 min and then cooled to room temperature. The extract was filtered through Whatman No.1 filter paper, and the filtered extract was stored in a freezer at 4 °C for further analysis. The schematic representation of the growth mechanism of the formation of Ag QDs/g-C_3_N_4_ structures under the sonochemical method is depicted in Figure 1. The growth of the silver QDs controlled by biomolecules was presented in ginseng extract under the sonochemical process. In the sonochemical process, the stacks of g-C_3_N_4_ were exfoliated and silver seeds were grown on the surface of the g-C_3_N_4_ structures. 

### 2.2. Characterization

The crystal structures of the prepared samples were characterized by X-ray diffraction (XRD; PANalytical X’pert diffractometer, Malvern, UK), using the CuKα_1_ radiation of wavelength λ = 1.5477 nm scanned in the 2θ range of 20–90 at 40 kV and 30 mA. The morphology analysis was carried out based on scanning electron microscopy (SEM; Hitachi S-4800, Tokyo, Japan) and high-resolution transmission electron microscopy images were recorded by a transmission electron microscope (TEM; Tecnai G2 F20 S Twin, 200 kV; FEI, Hillsboro, OR, USA). The oxidation state and the elemental and chemical composition were analyzed via X-ray photoelectron spectroscopy (XPS; Thermo Scientific; Waltham, MA, USA). The ultraviolet-visible diffuse reflectance spectra of prepared samples were recorded on a UV-Vis-NIR spectrophotometer (UV-vis-NIR DRS, Cary 5000; Agilent, Santa Clara, CA, USA). The functional group and structural vibrations were determined by Fourier transform infrared spectroscopy (FTIR, Thermo Scientific; Waltham, MA, USA). For hydrogen production experiments from water splitting, 5 mg of the prepared sample was added to a 5% lactic acid/water solution and dispersed. The mixture was kept under the light source for irradiation in an ambient atmosphere. The light source used a 300 W rated xenon lamp with a light intensity of 50 W/cm^2^, and the wavelength λ of the emitted light was greater than 400 nm. Furthermore, the generated hydrogen in the reactor was collected with an air-lock syringe and measured using a gas sensor chromatograph (YL-6500 GC system).

### 2.3. Photocatalytic Dye Degradation

To determine the photocatalytic performance, prepared photocatalysts were examined with a pollutant model (rhodamine B; RhB; 5 ppm) under visible light irradiation (visible light: 100 Watts). The photocatalysts (20 mg) were dispersed in a pollutant-containing solution (100 mL) with continuous stirring under light irradiation. The processes of degradation of the pollutants were measured with UV-Vis spectroscopic analysis. 

### 2.4. Antibacterial Activities

The prepared materials were examined for their medicinal values with preliminary studies, including antimicrobial studies with the microbes of *Escherichia coli*, *Bacillus subtilis*, *Listeria monocytogenes*, and *Staphylococcus aureus* using the disc diffusion method. We spread 100 µL of overnight bacterial cultures on Petri plates prepared with 20 mL of sterile LB agar (LBA, Himedia, Mumbai, India). We dissolved 10 µL of 0.1 mg/mL prepared NPs in DMSO, which were utilized to test the microbial inhibition potential. The anti-microbial activities of the compounds were compared with chloramphenicol at the same concentration. The inhibition zone diameter (mm) was measured using Vernier calipers after incubating the bacteria for 12 h at 37 °C [28].

## 3. Results and Discussion

**Figure 2:**
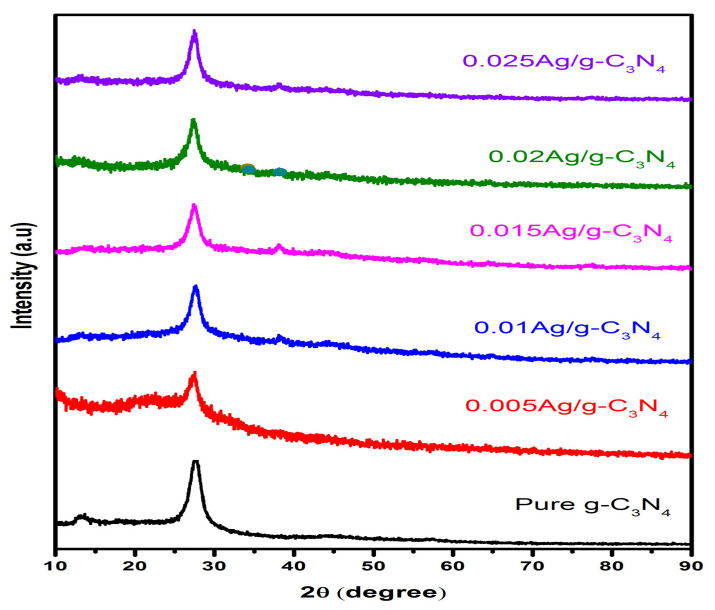
X-ray diaphragms of g-C_3_N_4_ and Ag-loaded g-C_3_N_4_ composites.

The XRD analysis was used to examine the phase purity and crystalline nature of prepared samples. The XRD patterns of pristine and Ag QDs anchored to g-C_3_N_4_ composite structures are depicted in Figure 2. The obtained diffractograms of the g-C_3_N_4_ sample showed peaks at 13.2° and 27.6° for the (100) and (002) diffraction planes attributed to the in-plane structural packing motif of tri-s-triazine and layered staking of the conjugated aromatic system, which is well-matched with standard data (JCPDS 87-1526). After the loading of Ag quantum dots to g-C_3_N_4_, the intensity of (100) peaks decreased, owing to the g-CN being covered by silver. After anchoring of Ag QDs to g-C_3_N_4_ 2D structures, the silver peaks were observed and results were matched with JCPDS no:87-0717. Moreover, the crystallite size of the particles was estimated to be 11.2 nm by using Scherrer’s equation: D = Kλ/βcosθ. In addition to that, the silver content increased as the silver diffraction peaks became stronger. Furthermore, no extra diffraction peaks were observed, which reveals that the absence of impurities and purity of Ag/g-C_3_N_4_ composites remained the same. 

**Figure 3:**
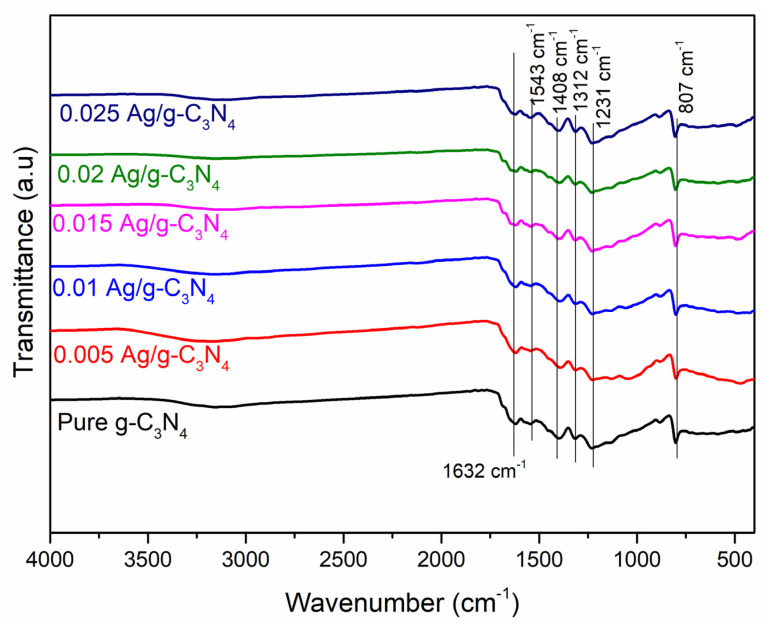
Fourier transform infrared spectra of g-C_3_N_4_ and Ag-loaded g-C_3_N_4_ structures.

The presence of functional groups at the surface of the prepared samples, determined through the Fourier transform infrared spectra (FTIR), was studied in the range of 4000 to 400 cm^−1^; the obtained results are presented in Figure 3. The FTIR spectrum of the pristine g-C_3_N_4_ was observed as broad absorption band at 1632 cm^−1^. The stretching vibration modes in the range of 3200–3000 cm^−1^ were due to the bonds that result from incomplete condensation of amino groups [29]. The peak at 1543 cm^−1^ was observed due to the Ag chelate band. The sequence of bands was observed in the range of 1500–1231 cm^−1^, which represents the starching modes of CN groups. The peaks were observed at 885 and 807 cm^−1^, corresponding to the deformation mode of N-H bonds and the breathing mode of tri-s-triazine units, respectively. Hence, the structure of g-C_3_N_4_ remains intact after the growth of Ag nanoparticles on the Ag-g-C_3_N_4_ composites. 

**Figure 4:**
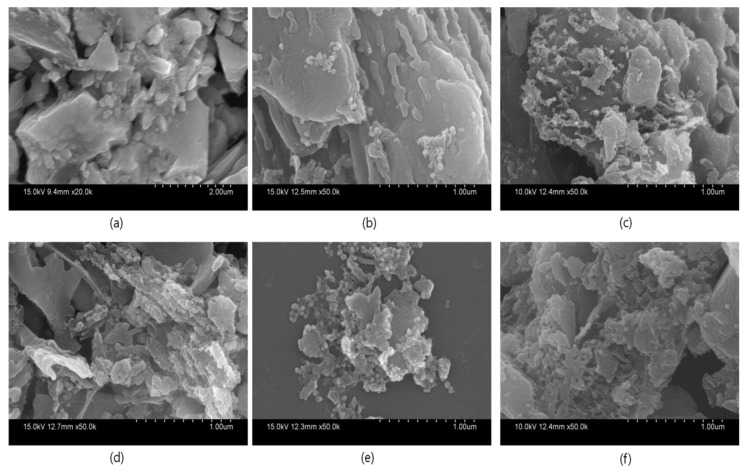
Scanning electron microscopy images of pure and Ag-loaded g-C_3_N_4_ structures (**a**) g-C_3_N_4_ and (**b**) 0.005 Ag/g-C_3_N_4_ (**c**) 0.01 Ag/g-C_3_N_4_ (**d**) 0.015 Ag/g-C_3_N_4_ (**e**) 0.02 Ag/g-C_3_N_4_ and (**f**) 0.025 Ag/g-C_3_N_4_ structures.

**Figure 5:**
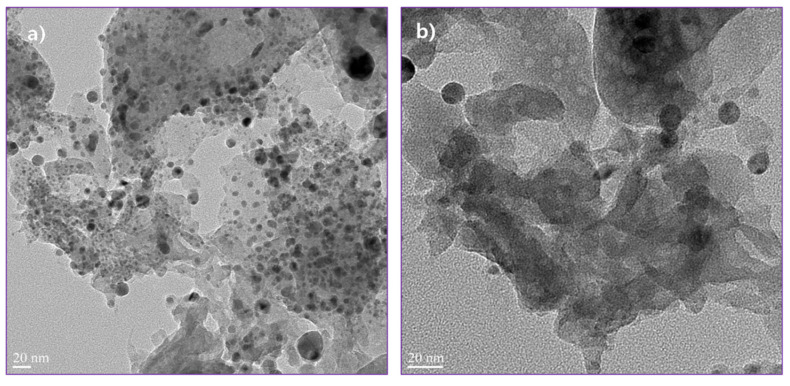
Transmission electron microscopy images of 0.02 Ag/g-C_3_N_4_ structures, (**a**) low magnification and (**b**) high magnification.

The morphological features of pure g-C_3_N_4_ with layer structures and Ag QDs anchored to g-C_3_N_4_ were studied with SEM analyses; the obtained results are shown in Figure 4. From the SEM images, the exfoliated g-C_3_N_4_ is represented in Figure 4a. For Ag-g-C_3_N_4_ samples, the content of silver increases the density of silver particles increased on the surface of the g-C_3_N_4_ structures (Figure 4b–f). For better understanding, the optimized sample was studied through transmission electron microscopic analysis. The TEM images of Ag-C_3_N_4_ were presented in Figure 5. The micrographs show that Ag particles were well spread out on the surface of the g-C_3_N_4_ sheets. The images illustrate that the Ag/g-C_3_N_4_ samples were nearly spherical shaped and that Ag particles were in order of less than 7 ± 2 nm in size. The size and shape of the particles plays a particularly significant role in chemical applications such as catalysis, owing to their high surface-to-volume ratio, which enables more surface-active sites, resulting in an enhanced rate of reaction. 

**Figure 6:**
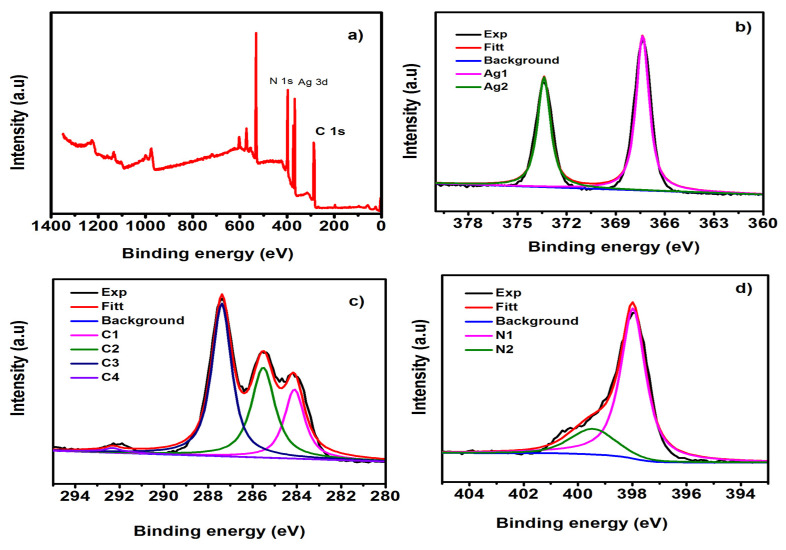
X-ray photoelectron spectra of 0.02 Ag/g-C_3_N_4_ structures (**a**) survey scan, (**b**) Ag 3d, (**c**) C 1s, and (**d**) N 1s elements.

XPS spectra of the Ag-g-C_3_N_4_ composite were recorded to analyze the chemical state and chemical composition of elements; the obtained results are depicted in Figure 5. The survey scan spectrum of the Ag-g-C_3_N_4_ composite was recorded in the range of 0-1350 eV. The characteristic peaks reveal the presence of elements C, N, and Ag, which were present in the prepared sample depicted in Figure 6a. The broad spectra of Ag 3d depicted two peaks at 367.3 and 373.3 eV, which are the binding energies of Ag 3d_5/2_ and Ag 3d_3/2,_ of Ag^+^, respectively, as shown in Figure 6b. Figure 6c reveals the deconvoluted spectrum of carbon and consists of the three binding energy peaks centered at 287.3, 285.4, and 284.1eV, ascribed to the tertiary carbon N=C–N_2_ in the g-C_3_N_4_ lattice, C–O from the adsorbed CO_2_ and external carbon contamination, respectively. Figure 6d shows the deconvoluted N 1s spectra, which was found to have four peaks that are attributed to the π-excitation at 404.1 eV, uncondensed terminal amino groups at 400.5 eV, tertiary nitrogen N atoms C_3_N (399.5 eV), and the sp-bond amino groups C_2_N–H involved in triazine rings (397.9 eV), respectively [30]. 

**Figure 7:**
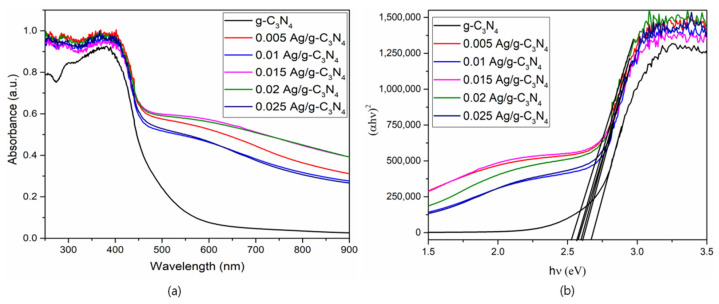
(**a**) UV-Vis-DRS properties of pristine and Ag/g-C_3_N_4_ structures (**b**) bandgap values of O- Ag/g-C_3_N_4_ structures.

To examine the optical properties of synthesized photocatalysts, the UV-DRS spectra of g-C_3_N_4_ and Ag-loaded g-C_3_N_4_ samples were determined and are illustrated in Figure 7. It was observed that all of the prepared photocatalysts had vigorous absorption capacity in the visible region, accredited to a charge-transfer transition between the Ag species and the g-C_3_N_4_. The bandgap values of pure g-C_3_N_4_, 0.005 Ag-g-C_3_N_4_, 0.01 Ag-g-C_3_N_4_, 0.015 Ag-g-C_3_N_4_, 0.02 Ag-g-C_3_N_4_, and 0.025 Ag-g-C_3_N_4_ were calculated and the bandgap values were found to be 2.67, 2.6, 2.59, 2.56, 2.51, and 2.53 eV, respectively. The values of the bandgap decreased noticeably after increasing Ag concentration, compared to that of the pure g-C_3_N_4_.

**Figure 8:**
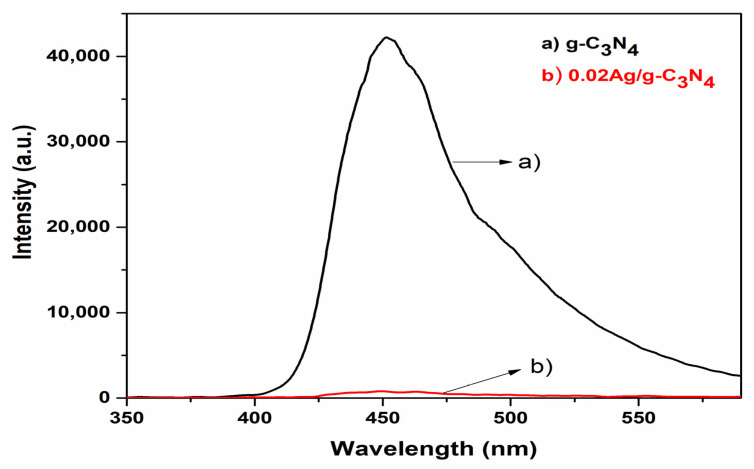
Typical emission spectrum of pure g-C_3_N_4_ and 0.02Ag/g-C_3_N_4._

To examine the recombination process, the charge carrier’s migration and the separation efficiency of photogenerated electron-hole pairs of the pure g-C_3_N_4_ and Ag/g-C_3_N_4_ composites, the photoluminescence emission (PL) spectra were recorded and are shown in Figure 8. Generally, a high PL emission intensity is due to the significant recombination of electron-hole pairs. For the pure g-C_3_N_4_ and Ag-C_3_N_4_ composites, the intensity peak was observed in the visible region at 452 nm. Nonetheless, the emission intensity of Ag/g-C_3_N_4_ was observed as lower than that of the g-C_3_N_4_ sample, suggesting that the rate of electron-hole pair recombination was suppressed in the Ag/g-C_3_N_4_ composite.

**Figure 9:**
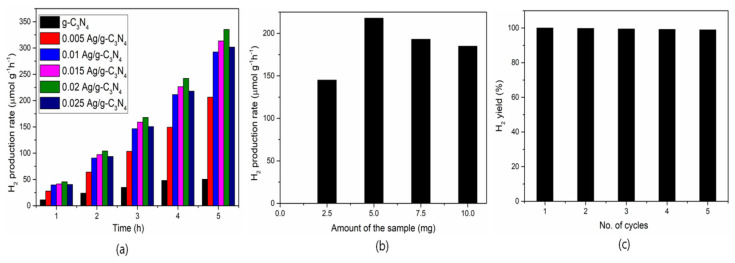
(**a**) Hydrogen production rate with the function of time for the pure g-C_3_N_4_ and Ag-anchored g-C_3_N_4_; (**b**) Amount of hydrogen production with different amounts of catalyst, and (**c**) reusability of catalyst.

The rate of H_2_ production in an aqueous solution for g-C_3_N_4_, 0.005 Ag-g-C_3_N_4_, 0.01 Ag-g-C_3_N_4_, 0.015 Ag-g-C_3_N_4_, 0.02 Ag-g-C_3_N_4,_ and 0.025 Ag-g-C_3_N_4_ was examined; the results are depicted in Figure 9a. The H_2_ evolution was monitored every 1 h for 5 h under visible light irradiation for prepared pristine and composite structures. In order to explore the influence of Ag QDs on photo**c**atalytic H_2_ evolution, different amounts of silver content in g-C_3_N_4_ were evaluated; the results are presented in Figure 9a. 

To explore the influence of Ag on photocatalytic H_2_ generation, a series of experiments were performed on the different concentrated Ag-loaded g-C_3_N_4_ composites under controlled conditions of identical irradiation. The loading of Ag in the composites significantly increased the production of H_2_ from the lactic acid aqueous solution. Furthermore, the amount of H_2_ that evolved increased proportionally with prolonged reaction time under the catalyst. Among prepared photocatalysts, the 0.02 Ag-g-C_3_N_4_ composite showed a significantly high rate of H_2_ evolution among all other catalysts. The highest and lowest rate of H_2_ evolution for 0.02 Ag-g-C_3_N_4_ and 0.005 Ag-g-C_3_N_4_ was 335 μmol h^−1^ and 45 μmol h^−1^, respectively. A high rate of H_2_ production was observed for the 0.02 Ag-g-C_3_N_4_ composite, owing to the efficient photocatalytic activity due to a greater number of H^+^ ions being adsorbed on the large surface. Additionally, the layers of g-C_3_N_4_ were exfoliated, which enabled a high surface area under the sonochemical process, resulting in a high rate of H_2_ production. Moreover, the amount of catalyst is one of the important parameters to optimize the sample amount. The decrease in H_2_ evolution alongside increasing Ag content in g-C_3_N_4_ could be attributed mainly to the development of a turbid colloidal substance, which restricts the absorption of exposed light by hampering the light path of the catalyst. Furthermore, there are many reasons for the decrease in H_2_ evolution: i) the greater amount of Ag effectively shields the g-C_3_N_4_ from incident light that significantly affects the excitation of charge carriers; ii) a high concentration of Ag hinders the active reaction sites in g-C_3_N_4_, which affects the H_2_ generation. Therefore, the amount of Ag is important for maximizing the amount of H_2_ production [31,32]. Thus, the 0.02 Ag-g-C_3_N_4_ catalyst had the optimum amount of Ag for maximizing H_2_ evolution. Regarding the amount of photocatalyst loadings for 2.5, 5, 7.5, and 10 mg of 0.02 Ag/g-C_3_N_4_ in lactic acid, a water solution was determined; the obtained results are depicted in Figure 9b. Additionally, one more important feature is the lifetime of the sample to evaluate the catalyst performance and the obtained reusability results are presented in Figure 9c. The H_2_ production performance of the 0.02 Ag/g-C_3_N_4_ sample was slightly decreased after 5 cycles, which may be due to the lower surface interaction of reaction radicals in the lactic acid oxidation process, which resulted in the H_2_ production.

**Figure 10:**
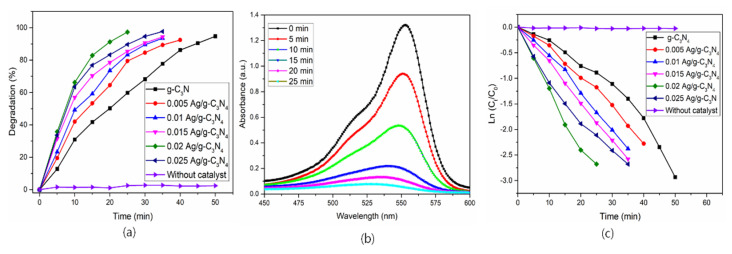
(**a**) Degradation of pollutant (RhB) under irradiation with prepared catalysts; (**b**) kinetics of the RhB with different intervals of irradiation time; (**c**) Ln Ct/C0 versus irradiation time.

The photocatalytic performance of prepared photocatalysts was analyzed and examined through a pollutant (Rhodamine B-RhB) model under visible light irradiation; the obtained results are shown in Figure 10. The efficiency in degradation of RhB in 25 min for the catalytic efficiency prepared photocatalysts was g-C_3_N_4_ (59%), 0.01-Ag/g-C_3_N_4_ (79%), 0.005-Ag/g-C_3_N_4_ (83%), 0.015-Ag/g-C_3_N_4_ (85%), 0.02-Ag/g-C_3_N_4_ (97%) and 0.025-Ag/g-C_3_N_4_ (89%). The 0.02-Ag/g-C_3_N_4_ (97 %) sample had a better performance on the rhodamine B degradation efficiency compared with all prepared samples. 

**Figure 11:**
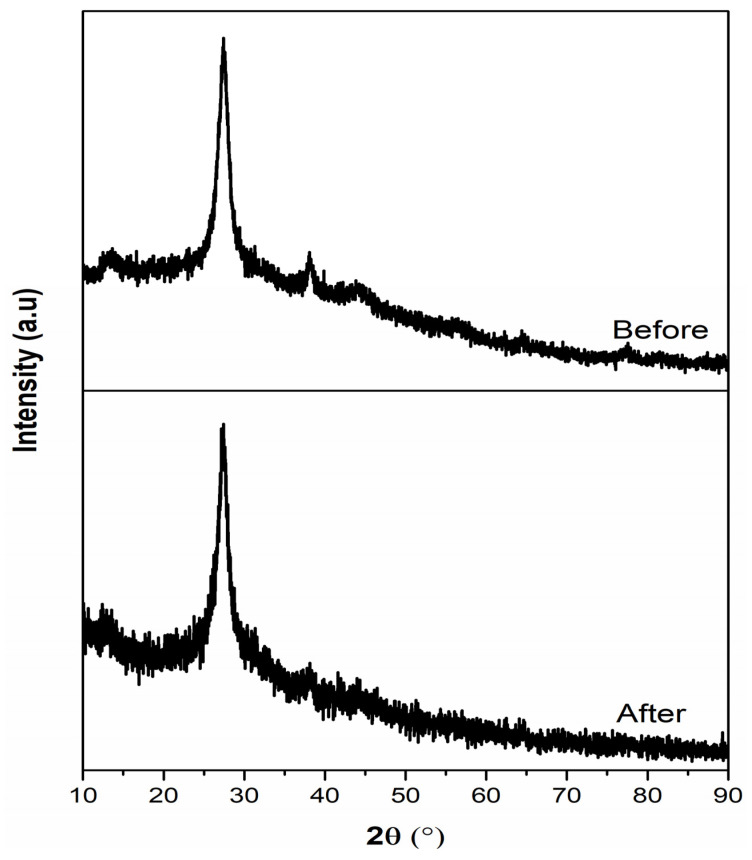
X-ray diffraction spectra of 0.02 Ag/g-C_3_N_4_ structures before and after dye degradation.

Moreover, the diminishment of the pollutant (RhB) was examined with UV-Vis absorption spectra under light irradiation, a maximum absorption peak was observed at 556 nm with the presence of 0.02 Ag/g-C_3_N_4_. The absorption spectra of RhB with different time intervals were represented in Figure 10b. The intensity of the absorption of the spectra was decreased with prolonged irradiation time, and it disappeared after 25 min of irradiation time, which strongly suggests that the pollutant was diminished from water. Moreover, in the reduction process of the pollutant (RhB), the absorption peak position slightly shifted towards the lower wavelength region, which may be due to the formation of intermediate compound demethylation and N-demethylation to small molecules and CO_2_ in the reduction process [33]. The efficiency of prepared photocatalysts was estimated by using the Langmuir–Hinshelwood kinetic model equation, ‒ln (C_t_/C_0_)=kt, where C_t_ and C_0_ are the concentration at irradiation and initial time, k is the apparent rate constant, and t is the irradiation time of light. The obtained results suggest that the synthesized pure and Ag decorated g-C_3_N_4_ samples show a good linear relationship and that the sample Ag/g-C_3_N_4_ (0.02) displays better results than the remaining prepared samples (Figure 10c). Moreover, Ag/g-C_3_N_4_ (0.02) exhibited the profound apparent rate constant of 1.45 × 10^−3^ s^−1^, which is higher than in the recent literature [34,35,36,37]. Moreover, there is no degradation of dye under light without a catalyst. Furthermore, the stability of the optimized catalyst was studied with XRD analyses, and is represented in Figure 11. There were no significant differences in XRD spectra, and there were no additional peaks observed, which suggests that the prepared catalysts are highly stable.

**Figure 12:**
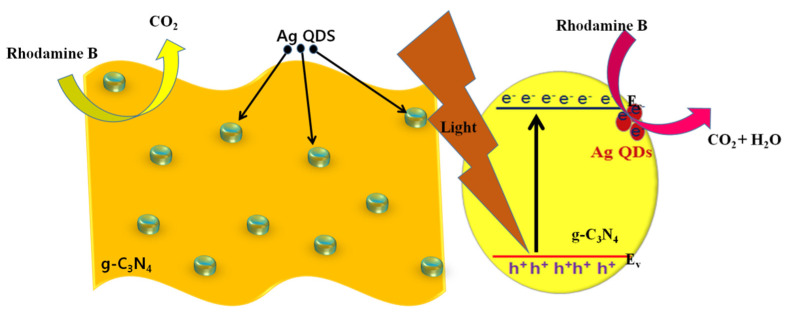
Schematic representation of the dye degradation mechanism under light irradiation.

The possible photocatalytic mechanism of prepared photocatalysts under visible light irradiation is depicted in Figure 12. The dimension of prepared catalysts plays a significant role in the catalytic application because the catalytic properties depend on the size and shape of the nanoparticles, which enables the enlarged surface atoms. Furthermore, Ag QDs were anchored to exfoliated g-C_3_N_4_ nanostructures, which enables the surface to volume ratio, the demise of the photogenerated electron-hole pair recombination rate, and the prolonged lifetime of the transmission of photoinduced electron-hole pairs. For the light irradiation on all prepared photocatalysts, the electrons were transferred from the silver to g-C_3_N_4_ and the photoinduced electrons from the valance band to the conduction band of g-C_3_N_4_; the generated electrons and holes underwent an oxidation and reduction process of pollutant degradation and water oxidation for H_2_ production. 

The preliminary examination of the prepared nanocomposites’ inhibition activity on selected microbes was as presented in Table 1, which shows that all the prepared nanocomposites have inhibition activity on the tested microbes. The highest inhibition was observed with optimized 0.02Ag/g-C_3_N_4_ on the selected microbes, *E. coli* (20 mm), *Bacillus* (18 mm), *S. aureus* (16 mm), and the compound 0.025 Ag/g-C_3_N_4_ showed the highest inhibition on *L. monocytogenes* (17 mm). The lowest activity was observed with pure g-C_3_N_4_ on *Bacillus* (10 mm) and *S. aureus* (10 mm) and the compound 0.001 Ag/g-C_3_N_4_ showed the lowest inhibition activity on *L. monocytogene* (10 mm). No activity was observed on *L. monocytogenes* by the pure g-C_3_N_4_. *L. monocytogenes* is a well known Gram-positive foodborne pathogen causing outbreaks in every country. In the present study, it was observed that four out of six nanocomposites acted against *L. monocytogenes* growth. From the results, it can be suggested that the antimicrobial activity is mainly due to the presence of silver, though the pure g-C_3_N_4_ also showed microbial inhibition at this low concentration. In addition, the highest activity was found in the optimized concentration (0.02 Ag/g-C_3_N_4_). The pattern of microbial inhibition is dose-dependent, and it is very clear that the inhibition is directly proportional to the concentration of silver. 

**Figure 13:**
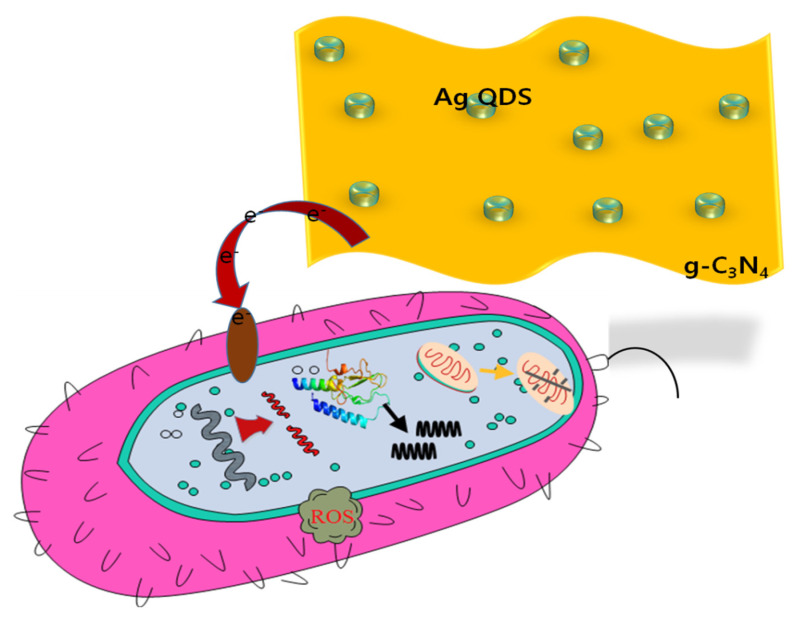
The hypothetical antimicrobial mechanism of Ag/g-C_3_N_4_ nanostructures.

The prepared nanocomposites actively inhibited the selected microorganisms. The activity may be because of their smaller size, large surface area, and stability. After dissolving the nanocomposites, the silver ions released from the composite penetrate the microbial membrane and disturb Ca^2+^ absorption. This caused cell membrane damage and leaking out of the intracellular ions. In addition, the electrostatic interactions by the adhesion of Ag/g-C_3_N_4_ NPs facilitate the release of silver ions and alter the microbial membrane structure [38]. The transformed cell membrane is more vulnerable to additional exchanges and to the diffusion of nanoparticles and leakage of the intracellular organelles. The proposed mechanism of action for the Ag/g-C_3_N_4_ compound is portrayed in Figure 13. The Ag/g-C_3_N_4_ composites showed efficient inhibition due to the combined effects of adhesion and penetration. Adhesion of microbes on the g-C_3_N_4_ mesh-like structure and penetration of Ag ions enhances the inhibition by intermingling with the electron transport and destroying the genetic material by breaking the phosphodiester bonds. Denaturation of the proteins and mitochondrial damage by oxidative stress due to the inorganic metal and metal oxide nanoparticles increases the chances of cell death. The important factors that affect the antimicrobial activity of nanoparticles are the size, shape, dosage, stability, and morphology of the nanoparticles, as well as the treatment time. The results are from the previous reports on the antimicrobial activity of Ag/g-C_3_N_4_ [38,39,40,41]. 

## 4. Conclusions

The composites of Ag QDs-decorated g-C_3_N_4_ structures were successfully prepared by the sonochemical method, and their bandgap was studied by UV-DRS analyses. The exfoliation g-C_3_N_4_ and anchoring of Ag QDs to exfoliated g-C_3_N_4_ is evidenced by the TEM analyses. The PL spectra strongly suggested that a decreases in the recombination rate of photogenerated charge carriers in Ag QDs/g-C_3_N_4_ structures. The significant enhancement in photocatalytic activity is due to the combination of Ag and g-C_3_N_4_ giving the reduction of the recombination rate of photoinduced electron-hole pairs. Moreover, it can be inferred that Ag and g-C_3_N_4_ in the system can play an important role in the improvement of photocatalytic activity for hydrogen production from water splitting and pollutant removal. The photocatalytic performance was examined through the RhB pollutant model under the irradiation of visible light. The maximum value of the degradation rate of RhB was noted for 25 min of visible radiation illumination for the Ag/g-C_3_N_4_ (0.02) sample. Moreover, the prepared materials performed the activity against the anti-bacterial property.

## Figures and Tables

**Table 1:** The performance of the antibacterial activities of prepared nanomaterials against different bacterial strains.

Compound	Zone of inhibition (activity) (mm)			
	*E. coli*	*B. subtilis*	*L. monocytogenes*	*S. aureus*
**a**	12	10	-	10
**b**	15	12	10	12
**c**	15	13	12	12
**d**	16	15	12	14
**e**	20	18	16	16
**f**	20	19	16	17
Chloramphenicol	22	24	22	20

**a**-pure g-C_3_N_4_; **b**-0.005Ag/g-C_3_N_4_; **c**-0.01Ag/ g-C_3_N_4_; **d**-0.15Ag/ g-C_3_N_4_; **e**-0.02Ag/ g-C_3_N_4_ and **f**-0.25Ag/g-C_3_N_4_.

## Data Availability

Data available on request due to restrictions.
